# Botanical Description of British Plants

**Published:** 1808-05

**Authors:** 


### *Botanical Description of British Plants.*

[ Continued from our last, pp. 353—361. ]

26. INULA. *I. hellenium. Hellenium. Enula campana. Aster.*

*Ang.* Elecampane.

*Gen. Desc.* Recept. naked. Down hair-like. Anthers with 2 bristles at the base.

*Spec. Desc. Leaves* embracing, egg-shaped, wrinkled, cottony underneath, deep green above, and slightly hairy, serrated. *Scales of cal.* egg-shaped. *Stem* 5 to 6 feet high, branched, scored, cottony. *Flowers* v. large, solitary, terminating, yellow: *flor. of circumf.* 1 to 1½ inch long, with 3 pointed teeth.—*Moist meadows.* BL. July, August.

*Use.* This is seldom to be met with in its wild state, but is commonly cultivated in gardens, from whence the shops are supplied with the root, which is the part directed for medicinal use. When the root is thoroughly dried and kept for a length of time, its smell becomes much stronger and more grateful, approaching then very near to the odour of the Florence orris. Its taste on first being chewed is glutinous and somewhat rancid, quickly succeeded by an aromatic bitterness and pungency. It was held in very high estimation by the ancients, *(see Schroder p.* 602. and *Alston's M. M. i.* 454.*)* Bergius also ascribes many virtues to the root; and from its sensible and chemical qualities, it promises to be a medicine of some efficacy: but in the diseases in which it is principally recommended, there is no satisfactory evidence of its medicinal powers, viz. dyspepsia, pulmonary affections, and uterine obstructions. *See Cullen M. M. ii.* 459. One drachm of the root in infusion, and from 2 drs. to ½ an oz. in decoction is the dose usually given.—*Woodville.* The root is esteemed a good pectoral. Dr. Hill says he knows from experience that an infusion of the fresh root, sweetened with honey, is an excellent medicine in the hooping-cough. The rot in sheep is cured by a decoction of the root.—*Withering.* A conserve of the root is advised for asthmas and disorders of the breast and lungs, as good to promote expectoration. A decoction of it in water, an infusion of it in wine, or a spirituous extract, are extolled as stomachic and sudorific; and are prescribed in crudities of the stomach, indigestion, hypochondria, and contagious diseases. Outwardly applied, a decoction of it is said to

 cure cure the itch. Bruised and macerated in urine with balls of ashes and whortle berries it dyes a fine blue colour.—*Lightfoot.* Horses and goats eat it; cows, sheep, and swine refuse it.

27. INULA. *I. dysenterica.*

*Ang.* Middle elecampane. Flea bane.

*Gen. Desc.* As above.

*Spec. Desc.* *Leaves* embracing, heart-oblong, obscurely toothed, cottony. *Stem.* woolly, forming a kind of panicle, upright, cylind. scored. *Cal. scales* bristle-shaped, soft bowed back, coloured at the edge, numerous, cottony. *Flowers* yellow, terminating, solitary. *Recept.* rough with short, stiff, projecting, spear-shaped points. *Flor. of circumf.* with 3 teeth at the end. *Seeds* rough.—*Moist meadows, watery places.* BL. August, Oct.

*Use.* It has a peculiar smell, which has sometimes been compared to that of soap. The bloody flux, which to a great degree affected the Russian soldiers in an expedition to Persia under General Keit, was cured by the use of this plant. Horses and cows are not fond of it; sheep and goats refuse it.—*Withering.*

28. BELLIS. *B. perennis.*

*Ang.* Daisy.

*Gen. Desc.* Recept. naked, conical. Down 0. Cal. hemispherical, with equal scales. Seed inversely egg-shaped.

*Spec. Desc.* *Stalk* naked, one-flowered, hairy, solid at bottom, hollow at the end, sometimes with a few leaves. *Leaves* oblong blunt, notched, spread on the ground. *Flor. of circumf.* notched, bright white, tipt with fine carmine, pink beneath: *fl. of centre* yellow.—*Meadows and pastures.* BL. March, Sept.

*Use.* The leaves are slightly acrid; and the roots have a penetrating pungency: but no attention is paid to it, except what is claimed by the beauty of its flowers. The flowers close at night. Horses, sheep, and cows refuse it. *Linnæus.*

29. CHRYSANTHEMUM. *C. segetum.*

*Ang.* Corn marigold. Goulans. Goldins. Marigold goldins. Buddle.

*Gen. Desc.* Recept. naked. Down 0, but a kind of border. Cal. hemispherical, tiled; scales membranaceous at the edge.

*Spec. Desc.* *Leaves* embracing, jagged upwards, tooth-serrated towards the base, sea green, varying in figure, as wedgewedge-strap or spear-shaped. *Flowers* v. large, terminating, yellow. *Flor. of circ.* oval, ½ an inch long. *Cal. scales* oval, blunt, sea green. *Seeds* slightly serrated, whitish. *Plant* smooth.—*Corn-fields.* Bl. June, Oct.

*Use.* It is made use of in Germany for the purpose of dying yellow. It is an extremely troublesome weed in arable land, and being very common in Denmark, there is a law in that country obliging farmers to root it out of their fields. It may be destroyed by dunging the ground in autumn, followed by a summer fallow, and harrowing about five days after sowing the grain. Its yellow flowers, however, which follow the sun in a very remarkable manner, give a brilliancy to the fields in tillage, and please the eye of the passing traveller.—*Linn.* A large quantity, which grew on some arable land, was cut when in flower, dried, and eaten by horses as a substitute for hay.—*Withering.*

30. Matricaria. *M. parthenium.*—*M. vulgaris.*

*Ang.* Feverfew.

*Gen. Desc.* Recept. naked. Down 0. Cal. hemispherical, tiled; scales rather pointed, not skinny at the edge.

*Spec. Desc.* *Leaves* compound, flat, alternate, slightly hairy; *leafits* egg-shaped, deeply cut, 2 or 3 pair, a large one terminating three-lobed. *Fruit-st.* branched. *Flowers* solitary, sometimes double. *Flor. of circumf.* wedge-shaped, white; *of centre* yellow at the top, crooked, sprinkled with shining particles. *Stem* 3 feet high, firm, angular, scored, slightly hairy.—*Waste places, hedges, walls.* Bl. June, July.

*Use.* In natural affinity this plant ranks with camomile and tansy, and its sensible qualities show it to be nearly allied to them in its medicinal character. Its virtues are stated by Bergius to be tonic, stomachic, resolvent, and emmenagogue. It has been successfully given as a vermifuge, and for the cure of intermittents; but its use is most celebrated in female disorders, especially hysteria: in this disorder its efficacy, according to Sim. Paulli, was very remarkable. Its smell, taste, and analysis, prove it to be a medicine of considerable activity; and Murray remarks that it deserves more attention than is paid to it.—*Woodville.* It is esteemed a good emmenagogue, and useful in hysteric complaints: the best way of taking it, is in a slight infusion. It is also an agreeable carminative and bitter; it strengthens the stomach and disperses flatulencies. The expressed juice is used as a vermifuge. It has been likewise recommended as a febrifuge, hence its English name.—*Lightfoot.* It yields an essential oil by distillation. A horse refused it.—*Withering.*

31. Matricaria. *M. chamomilla.*

*Ang.* Chamomile feverfew.

*Gen. Desc.* As above.

*Spec. Desc. Recept.* conical, almost cylind. dotted. *Rays* expanding. *Cal. scales* equal at the edge, bluntly spear-shaped, hairy, membranaceous at the edge, a green line along the back. *Stem* scored, branched. *Leaves* doubly winged, or more than doubly, with slender segments. *Flowers* solitary, terminating: *fl. of circ.* white, strap-shaped; *of centre,* yellow. *Seeds* minute, pale brown, furrowed.

*Var. M. Suaveolens. Sweet-scented Feverfew.—In corn-fields, cultivated ground, road sides, dunghills.* Bl. May, August.

*Use.* In Finland an infusion of this is used in consumptive cases.—*Withering.* The flowers are reckoned antiseptic, and approach in quality to the Peruvian bark; 20 or 30 grs. of them readily promote sweat, and are recommended as a cure for the ague; mixed with salt of wormwood, they are said to be useful in fevers. A decoction of them is esteemed good in nephritic complaints, and are efficacious in assuaging the pains of the cholic and dysentery. Baths, clysters, and cataplasms of them are used also in the last intention. A blue essential oil is obtained from the flowers by distillation, and is supposed to contain all their virtues.—*Lightfoot.* Its properties resemble those of the Anthemis nobilis.—Cows, sheep, and goats eat it; horses are not fond of it; swine refuse it.

32. Anthemis. *A. nobilis.— Chamæmelum. Leucanthemum odoratius.*

*Ang.* Chamomile. Sweet-scented chamomile. Roman chamomile.

*Gen. Desc.* Recept. chaffy. Down 0. Cal. hemispherical, scales nearly equal, florets of the circumference more than 5.

*Spec. Desc. Leaves* winged, compound, strap-shaped, acute, somewhat woolly, wings distant; little wings two or three-cleft, pointed, hairy, greyish. *Stems* trailing, hairy. *Cal.* hairy, with broad, shining, membranaceous edges *Flowers* solitary; *fl. of circ.* oval, white; *of centre* yellow.—*Boggy, or sunny pastures.* Bl. August, Sept.

*Use.* Both the leaves and the flowers have a strong though not ungrateful smell, with a very bitter nauseous taste, but the latter are bitterer and considerably more aromatic: they give out their virtue both to water and rectified spirit, and when the flowers have been dried so as to be pulverable, pulverable, the infusions prove more grateful than when fresh, or but moderately dried. They possess the tonic and stomachic qualities usually ascribed to simple bitters, having very little astringency, but a strong odour of the aromatic and penetrating kind, from which they are also judged to be carminative, emmenagogue, and in some measure antispasmodic and anodyne: they have long been successfully used for the cure of intermittents, in which they were found by Morton, &c. more effectual than the Peruvian bark; and it was the opinion of Dr. Pitcairn that they were in this respect equal to the bark. They have been found equally useful in fevers of the irregular nervous kind, accompanied with visceral obstructions.—(*Pringle Dis. of the Army*, 216.) The use of camomile flowers as a substitute for Peruvian bark in the cure of intermittent fevers is authorized by the testimony of several respectable physicians; and Dr. Cullen says that by giving, agreeably to Hoffman's method, several times during the intermission, from half a drachm to 1 dr. of the flowers in powder, he has cured intermittents. But he found that these flowers when given in large quantity, readily run off by stool, defeating thereby the purpose of preventing a return of the paroxysms; and without joining them with an opiate or astringent, he says he could not commonly employ them, *M. M. ii.* 79. They have been found useful in hysterical affections, flatulent or spasmodic cholics, and dysentery; but from their laxative quality, they are said to be hurtful in diarrhœas. A simple watery infusion of them is frequently taken in a tepid state for the purpose of exciting vomiting, or for promoting the operation of emetics. Externally, they are used in the decoctum pro fomento, and they are an ingredient in the decoctum pro enemate.—*Woodville.* The powdered flowers, in large doses, have cured agues, even where the bark had failed. Both the leaves and flowers possess very considerable antiseptic properties, and are, therefore, used in antiseptic fomentations and poultices. From their antispasmodic powers, they are frequently found to relieve pain, either applied externally, or taken internally. In calculous cases, the flowers are recommended by Ray. An infusion of them is often used as a stomachic, and as an antispasmodic: in large quantities, it excites vomiting.—They afford an essential oil: this is probably secreted by the minute glands by which the tubes of the florets appear to be beset.—*Withering.*

33. ANTHEMIS. *A. tinctoria.*

*Ang.* Ox-eye chamomile.

*Gen. Desc.* As above.

*Spec. Desc.* *Leaves* doubly winged, serrated, cottony and sea-green underneath; *wings* spear-shaped, toothed, slightly hairy. *Stem* supporting a corymbus, scored, slightly hairy, much branched. *Fruit-st.* long, naked, scored, slightly hairy, terminating, one-flowered. *Bloss.* yellow. *Fl. of circ.* broad, 3 teeth.—*Sunny pastures, not common.* Bl. July, August.

*Use.* The flowers afford a remarkably clear and good yellow dye. The flowers of the chrysanthemum segetum resemble them very much in appearance, but experience proves that they cannot be substituted in their place. Horses and goats eat it; sheep are not fond of it; cows and swine refuse it.—*Withering.*

34. Achillea. *A. Ptarmica.*

*Ang.* Sneeze-wort. Yarrow. Goose-tongue. Bastard pellitory.

*Gen. Desc.* Recept. chaffy. Down 0. Cal. egg-shaped, tiled. Strap-shaped florets from 5 to 10.

*Spec. Desc.* *Leaves* strap-spear-shaped, embracing, finely serrated, upright, scattered, sitting, firm, smooth, dark green; ends of serratures white, of a bony hardness. *Stems* 1 to 2 feet high, firm, angular, smooth, often reddish. *Flor. of circumf.* oval, broad, 3 teeth, a short tube, rarely more than 12; *of cent.* numerous, short, dirty yellow. *Chaff.* woolly.—*Moist meadows, shady places.* Bl. July, August.

*Var. with double flowers.*

*Use.* The young tops are sharp and pleasant in spring sallads. The roots have a hot biting taste. The powdered leaves excite sneezing. Horses, cows, sheep, goats, and swine eat it.—*Withering.*

35. Achillea. *A. millefolium.*—*Millefolium vulgare album. M. terrestre.*

*Ang.* Yarrow. Milfoil yarrow. Nosebleed.

*Gen. Desc.* As above.

*Spec. Desc.* *Leaves* doubly winged; segm. of wings strap-shaped, toothed, woolly or hairy. *Stem* angular, cottony. *Flowers* in a corymbus, white, or reddish purple, sometimes nearly crimson: *fl. of circ.* 5, *border* nearly circular, bent back, slightly 3 cleft: *of centre* 15, or more; 4 or 5 only expanding at once. *Cal. scales* woolly, skinny at the edges.—*Meadows, pastures, road sides.* Bl. June, August.

*Use.*

*Use.* The leaves and flowers have a weak aromatic smell, and a bitterish, rough, somewhat pungent taste. This plant was esteemed by the Greeks an excellent vulnerary and styptic. Instances of its good effects in this way, in hæmoptysis, epistaxes, menorrhagia, and hæmorrhois, are likewise mentioned by several of the German physicians, especially Stahl and Hoffman, whore commend it also as an efficacious remedy in various other diseases; the former found it not only an astringent, but a powerful tonic, antispasmodic, and sedative: in proof of the last mentioned quality, it may be remarked that in Sweden it is used in making beer, in order to render the liquor more intoxicating; it is employed, according to Sparrman, for the same purpose in some parts of Africa. The leaves and flowers are both directed for medicinal use in the Edinb. Pharm. but in the present practice, this plant is disregarded.—*Woodville.* The fresh young roots have a glowing warm taste, approaching to that of the Contrayerva, for which Dr. Grew thinks they might be substituted. *On tastes, c. v.* § 2. This plant has an astringent quality, and is reckoned good to stop all kinds of hæmorrhages, and to heal wounds, but is now out of use. In Scotland, however, the highlanders still use an ointment made of it, to heal and dry up wounds. To cure the head-ache, the common people sometimes thrust up their nostrils a leaf of it, for the purpose of making the nose bleed; an old practice, the origin of one of its English names. In the Swedish province of Dalecarlia, it is used instead of hops to mix with ale, and it gives an intoxicating quality to the liquor.—*Lightfoot.* The expressed juice of the yonng tops is said to be very serviceable in the piles: a table spoonful being taken in a little new milk night and morning for a month or more, with the interval of about a week after every four.—*B.* The leaves are celebrated by medical writers for a variety of purposes, but are little attended to. The flowers yield an essential oil. Sheep and swine eat it; horses, cows, and goats are not fond of it.—*Withering.*

( To be continued. )

